# Short-term behavioural impact contrasts with long-term fitness consequences of biologging in a long-lived seabird

**DOI:** 10.1038/s41598-020-72199-w

**Published:** 2020-09-14

**Authors:** Natasha Gillies, Annette L. Fayet, Oliver Padget, Martyna Syposz, Joe Wynn, Sarah Bond, James Evry, Holly Kirk, Akiko Shoji, Ben Dean, Robin Freeman, Tim Guilford

**Affiliations:** 1 Department of Zoology, University of Oxford, Oxford, UK; 2 School of Ocean Sciences, Bangor University, Bangor, UK; 3 Interdisciplinary Conservation Science Group, RMIT University, Carlton, Australia; 4 Graduate School of Life and Environmental Sciences, University of Tskuba, Tskuba, Japan; 5 Institute of Zoology, Zoological Society of London, London, UK

**Keywords:** Animal behaviour, Behavioural ecology

## Abstract

Biologging has emerged as one of the most powerful and widely used technologies in ethology and ecology, providing unprecedented insight into animal behaviour. However, attaching loggers to animals may alter their behaviour, leading to the collection of data that fails to represent natural activity accurately. This is of particular concern in free-ranging animals, where tagged individuals can rarely be monitored directly. One of the most commonly reported measures of impact is breeding success, but this ignores potential short-term alterations to individual behaviour. When collecting ecological or behavioural data, such changes can have important consequences for the inference of results. Here, we take a multifaceted approach to investigate whether tagging leads to short-term behavioural changes, and whether these are later reflected in breeding performance, in a pelagic seabird. We analyse a long-term dataset of tracking data from Manx shearwaters (*Puffinus puffinus*), comparing the effects of carrying no device, small geolocator (GLS) devices (0.6% body mass), large Global Positioning System (GPS) devices (4.2% body mass) and a combination of the two (4.8% body mass). Despite exhibiting normal breeding success in both the year of tagging and the following year, incubating birds carrying GPS devices altered their foraging behaviour compared to untagged birds. During their foraging trips, GPS-tagged birds doubled their time away from the nest, experienced reduced foraging gains (64% reduction in mass gained per day) and reduced flight time by 14%. These findings demonstrate that the perceived impacts of device deployment depends on the scale over which they are sought: long-term measures, such as breeding success, can obscure finer-scale behavioural change, potentially limiting the validity of using GPS to infer at-sea behaviour when answering behavioural or ecological questions.

## Introduction

Biologging is a powerful tool for understanding the behaviour and ecology of animals. Individuals are now routinely fitted with miniaturised data loggers capable of recording a wealth of information, including location, acceleration, heart rate, dive depth, and temperature. As the power to size ratio of devices continues to increase, it is now routine for multiple devices to be deployed simultaneously (e.g.^1^) and on ever-smaller animals (including insects^2^) in a variety of environments^3^. However, while the wide-scale usage of these devices has provided considerable insights into behaviour, movement, and physiology (e.g.^4–9^) it also has the potential to affect the tagged animal’s behaviour and hereby distort or bias the results of studies. As such, understanding the behavioural impacts of tagging is critical.

This, however, is challenging. While the great advantage of remote biologging is that we can record data about the biology of animals without direct observation, this also makes it difficult to test whether the devices themselves change animal behaviour, and therefore to assess whether the behavioural and ecological data collected are unbiased and representative. Meta-analyses of device effects have repeatedly found that tag deployment can lead to significant negative impacts on animals relating to survivorship, reproduction and activity^10,11^. In particular, behavioural impacts are widespread and phylogenetically diverse, and include impairments in locomotory ability (Yuma myotis bats *Myotis yumanensis*^12^; sharks^13^; bottlenose dolphins *Tursiops truncatus*^14,15^; grey partridge *Perdix perdix*^16^), increases in energetic expenditure (fish^17^; great cormorants *Phalacorax carbo*^18^), alterations to social behaviour (bald ibis *Geronticus eremita*^19^), increased autopreening (gyrfalcons *Falco rusticolus*^20^; African penguins *Spheniscus dermsus*^21^) at the expense of other behaviours such as feeding (Barrow’s goldeneye *Bucephala islandica*^22^), and reductions in general activity levels (house mouse *Mus musculus*^23^; freshwater mussel *Margaritifera margaritafera*^24^), foraging effort (emperor penguins *Aptenodytes forsteri*^25^), and nest attendance (Atlantic puffins *Fratercula arctica*^26^; alcids^27^). The impacts of tagging procedures vary across and within taxa, meaning that rather than employing a general measure of impact across all types of study, researchers must consider in detail the impacts of carrying a device on the specific species of interest in the context of the experimental design. When studying the behaviour of animals, effects such as these can have important consequences for the validity of the data collected. These may have important implications for behavioural studies or the interpretation of results, yet remain poorly understood.

Despite this, many authors neglect to consider fully the impacts that their devices may have. Though 80% of authors do make some reference to impacts of their tagging procedures, only 42% of these directly compare untagged and tagged individuals^10^. Increasingly, authors use breeding success to determine the effects of their tagging protocols^27–31^. However, this may be blunt. Devices may have more subtle and complex effects on behaviour which don’t manifest in changes in breeding success. For example, individual plasticity may allow individuals to buffer against the effects of costs of tagging in a way that preserves their reproductive success. Handicapping studies demonstrate that alterations to behaviour may not necessarily manifest in costs to the offspring if these costs are absorbed by the handicapped individual (e.g. great tits *Parus major*^32^; burying beetles *Nicrophorus vespilloides*^33^, its partner (e.g. northern flickers *Colaptes auratus*^34^, or some combination of the two (e.g. pied flycatchers *Ficedula hypleuca*^35^). Furthermore, single fitness-based measures are often unable to capture complex, long-term impacts on individuals. Negative effects of tagging on breeding success may not manifest until the years following the procedure (e.g. king penguins *Aptenodytes patagonicus*^36^), may cause reductions in return rates (e.g. adelie penguins *Pygoscelis adeliae*^37^), or increases in divorce (e.g. thick-billed murres *Uria lomvia*^38^. Yet in spite of mounting evidence that breeding success may not always be an appropriate measure of impact, particularly for behavioural studies, only 26% of published tagging studies in which impact is reported consider behavioural effects^11^.

A recent meta-analysis of the impacts of device deployments on a range of species has revealed significant effects of deployment on reproductive success, foraging behaviour, and survival^11^. In the Manx shearwater, a small diving seabird of the order Procellariform, hatching success has been previously reported as unaffected by device deployment^39^, but effects on behaviour have not been investigated. Here, we draw a multifaceted approach to determine the impact of device deployment on Manx shearwaters. Specifically, we investigate the effects of tracking on several behavioural measures in addition to breeding success. Our approach compares short- and long-term measures, across different combinations of devices, and between untagged and tagged animals, to determine precisely the effects of carrying a device for this species. We expected to find that device deployment does affect the behaviour of Manx shearwaters, with larger back-mounted devices having a greater effect than small leg-fitted ones^10^, and that these effects differ between incubation and chick-rearing because of the differing demands of these two periods on the breeding adults^40,41^. By comparing the impacts arising in measures of behaviour and fitness, we demonstrate that in this species, behavioural changes associated with instrumentation do not necessarily translate into altered fitness, meaning changes to one do not necessarily inform on the other.

## Results

In total, 591 foraging trips were extracted for subsequent analyses, representing 405 individuals from 174 nests. To ensure that all our measures were fully comparable, we subset these trips to include only those individuals for which overall breeding success and, if applicable, measures of chick provisioning, were known. From this, 171 foraging trips, representing 130 individuals from 72 nests, remained. All ensuing analyses were performed on both the complete and comparative datasets; effect sizes and significance values did not differ depending on which dataset was used. For brevity, only results from the subset comparative dataset are presented here. Results from analyses conducted on the complete dataset are reported in supplementary materials. Figures include data from the entire dataset.

The starting masses for birds in each deployment group did not significantly differ (no device: 388.8 ± 9.2 g, GLS: 405.3 ± 7.3 g, GPS: 397.4 ± 6.2 g, combined: 402.7 ± 3.9 g; χ^2^ = 4.0, df = 3, p = 0.264). Given these mean starting masses, the percentage body mass of each device equated to 4.8 ± 0.04% for the combined deployment, 4.3 ± 0.06% for the GPS-only deployment and 0.7 ± 0.08% for the GLS deployment. The metal ring carried by all birds equated to 0.2 ± 0.1% of body mass.

### Effects of tagging on breeding success

Deployment type did not significantly predict breeding success in the year of deployment (χ^2^ = 0.42, df = 3, p = 0.94) or the subsequent year (χ^2^ = 3.09, df = 3, p = 0.38). A breakdown of the percentage of eggs surviving until at least late-stage chicks is shown in Table 1.

**Table 1:** Percentage eggs resulting in late stage chicks for each deployment group in the year of deployment (*t*) and the year following deployment (*t* + *1*).

	No device	GLS	GPS	Combined
t	73.8%, *n* = 42	75.6%, *n* = 42	80.0%, *n* = 30	66.7%, *n* = 63
t + 1	72.3%, *n* = 26	63.6%, *n* = 33	84.2%, *n* = 19	76.5%, *n* = 34

*n* = number of eggs recorded in each group.

### Effects of tagging on foraging behaviour

**Figure 1:**
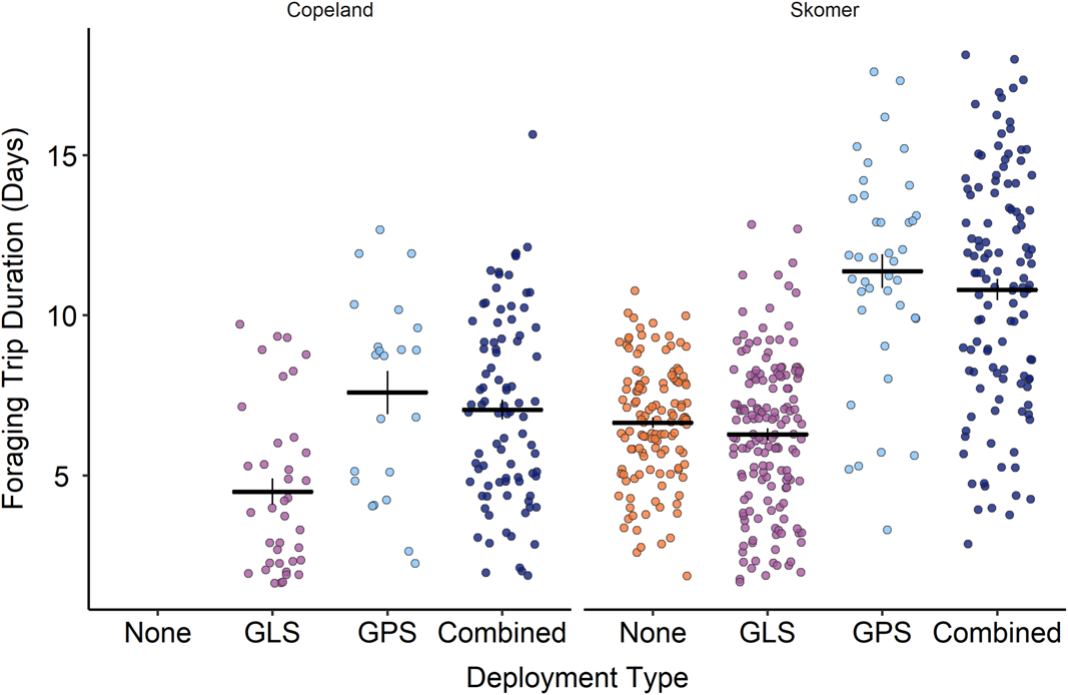
Foraging trip duration in days grouped by deployment type within island. Black horizontal lines indicate the mean for each group; black vertical lines indicate the standard error. Data are plotted with horizontal ‘jitter’.

Birds tracked on Skomer embarked on foraging trips during incubation which were significantly longer than those of birds on Copeland, in keeping with findings by Dean et al. (2015; Fig. 1; Copeland: 3.1 ± 1.2 days, Skomer: 9.1 ± 0.8 days; χ^2^ = 25.9, df = 1, p < 0.0001).

**Figure 2:**
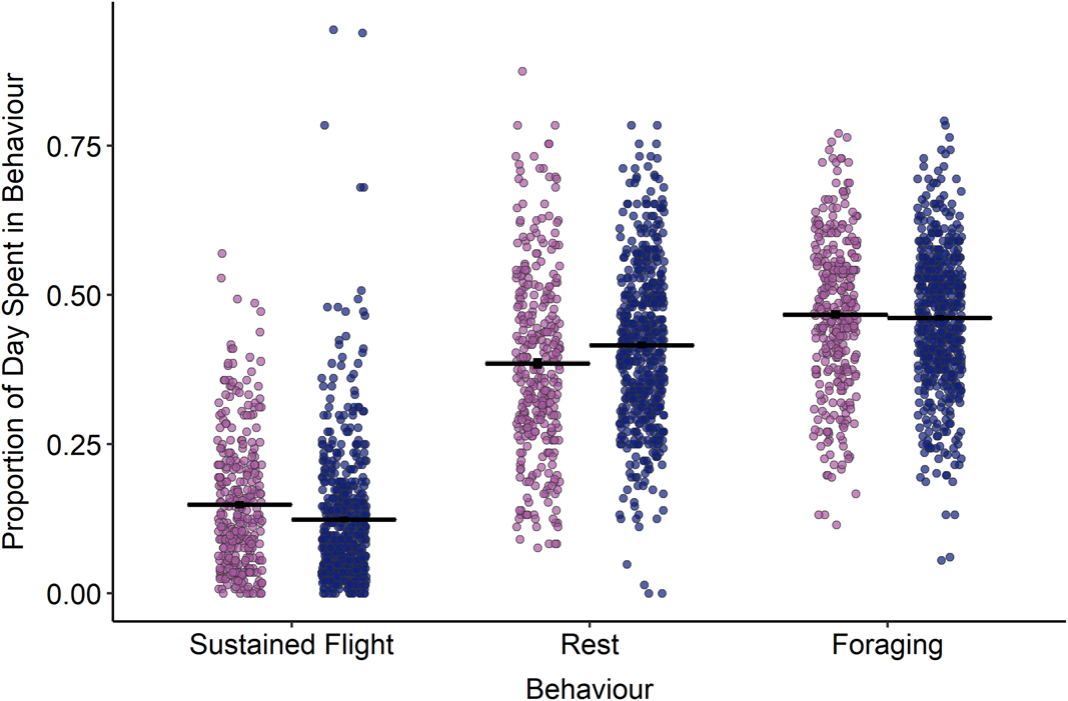
Proportion of each 24 hour period (00:00-23:59) spent in rest, flight, or foraging behaviour according to deployment type, where dark blue = combined GPS, and lilac = GLS only. Black horizontal lines indicate the mean for each group; black vertical lines indicate standard error. Data are plotted with horizontal ‘jitter’.

Birds carrying either a GPS or GPS in combination with a GLS embarked on foraging trips during incubation which lasted significantly longer than birds carrying a GLS only or no device (Fig. 1; no device: 4.7 ± 0.9 days, GLS: 5.5 ± 0.7 days, GPS: 9.8 ± 0.8 days, combined: 9.7 ± 0.6 days; χ^2^ = 60.0, df = 3, p < 0.0001; Table 2). There was no significant effect of the interaction of deployment type and starting mass (χ^2^ = 2.9, df = 3, p = 0.4). Compared to birds tracked with GLS only, on their foraging trips combined deployment birds spent a lower proportion of their day in flight (GLS: 0.18 ± 0.01, combined: 0.15 ± 0.02; χ^2^ = 6.0**,** df = 1, p = 0.01; Fig. 2). GLS and combined deployment birds did not differ in the proportion of their day dedicated to rest (GLS: 0.32 ± 0.02, combined: 0.35 ± 0.02; χ^2^ = 3.51 p = 0.08) or foraging (GLS: 0.35 ± 0.02, combined: 0.38 ± 0.03; χ^2^ = 3.1, df = 1, p = 0.7**).**

**Table 2:** Pairwise comparison of foraging trip duration during incubation for each deployment type. Significant values (p < 0.05) are in bold.

Device A	Device B	Mean diff (A − B)	t value	p
No device	GLS	− 0.8 ± 0.9	− 0.9	0.791
	GPS	− **5.1 ± 0.9**	− **5.1**	** < 0.0001**
	Combined	− **5.0 ± 0.8**	− **6.0**	** < 0.0001**
GLS	GPS	− **4.3 ± 0.9**	− **4.6**	** < 0.0001**
	Combined	− **4.2 ± 0.6**	− **6.5**	** < 0.0001**
GPS	Combined	0.1 ± 0.9	− 0.1	0.999

### Effects on foraging efficiency

**Figure 3:**
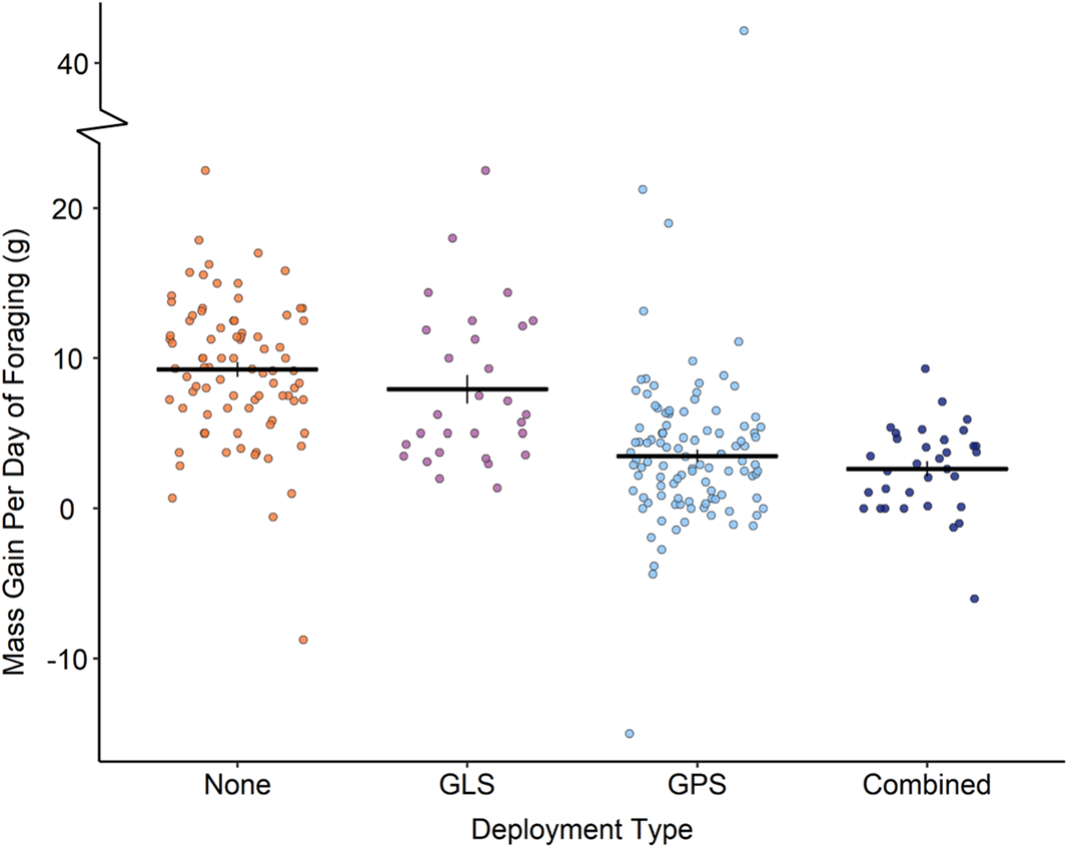
Mass gain per day (g) of the foraging trip during incubation according to deployment type. Black horizontal lines indicate the mean for each group, black vertical lines indicate standard error. Data are plotted with horizontal ‘jitter’.

Deployment type had a significant influence on the mass gained per day of the foraging trip during incubation (χ^2^ = 38.9, df = 3, p < 0.0001). Birds carrying GPS, whether or not in combination with a GLS, gained less mass per day of their foraging trip than GLS-tracked or untracked birds (no device: 9.0 ± 1.1 g, GLS: 9.1 ± 1.1 g, GPS: 2.5 ± 0.9 g, combined: 3.8 ± 0.7 g; Fig. 3). Considering the entire foraging trip, birds carrying GPS were found to gain less mass overall on their trips, despite their longer durations (no device: 55.5 ± 9.7 g, GLS: 64.8 ± 9.04 g, GPS: 28.9 ± 7.2 g, combined: 40.1 ± 5.8 g; χ^2^ = 12.3, df = 3, p = 0.006).

### Effects on chick provisioning

In total, the mass changes of 48 chicks were recorded over the years 2013–2017. Neither deployment of GLS or GPS during the provisioning period, nor deployment of GPS during incubation, had an effect on the duration of the provisioning period for chicks, the number of nights they were fed, the maximum mass they attained, or their average feed per night, relative to chicks of non-tracked parents (Table 3). During the tracking period itself, the average food delivery per nest visit did not differ between parents carrying different deployment combinations (no device: 29.3 ± 0.79 g, GLS: 29.1 ± 0.95 g, GPS: 30.4 ± 0.96 g, combined = 30.7 ± 1.0 g; df = 2, χ^2^ = 3.1, p = 0.379).

**Table 3:** Pairwise comparison of metrics of provisioning effort for each deployment type. P-values calculated relative to the ‘no device’ group.

Deployment	Nights fed		Peak mass (g)			Average feed (g)			Provisioning days	
	Mean	p-value	Mean	p-value		Mean	p-value		Mean	p-value
No device	29.3 ± 1.7	–	543.1 ± 13.9	–		29.3 ± 1.2	–		51.0 ± 3.3	–
GLS	28.8 ± 1.7	0.979	542.3 ± 14.5	1.000		29.4 ± 1.3	1.000		49.9 ± 3.3	0.862
GPS	29.4 ± 1.7	1.000	552.9 ± 13.6	0.927		30.5 ± 1.2	0.908		50.9 ± 3.3	1.000
GPS (incub)	29.2 ± 1.7	1.000	557.9 ± 18.2	0.896		30.5 ± 1.2	0.976		51.2 ± 3.3	0.999
Combined	29.4 ± 1.7	1.000	545.4 ± 13.9	0.999		30.7 ± 1.2	0.908		52.0 ± 3.3	0.903

Nights fed = total number of nights in which a provisioning visit was detected; peak mass = maximum mass attained by the chick; average feed = mean feed per night where provisioning visit detected; provisioning days = total number of days from hatching until last visit detected.

## Discussion

In this study we compared behavioural and fitness measures of tagging impact in a free-ranging breeding seabird. We found that the short-term deployment of GPS tags on Manx shearwaters is associated with significant foraging behavioural changes during incubation, but that these do not translate into reduced breeding success in either the year of tagging or the subsequent year. Thus, the perceived impact of tag deployment for this species may depend on the measure used to assess it, potentially leading to substantial effects on behaviour being overlooked. More specifically, during incubation, shearwaters which carried a GPS doubled their foraging trip durations, reduced flight time, and gained less mass per day of their trips. However, during chick rearing, no effects on parental provisioning could be observed: pairs in which one adult was tagged provisioned as much and as often as unmanipulated pairs. Thus, breeding success may be insufficient to capture short-term alterations in behaviour.

There are several mechanisms by which the attachment of the GPS tag could have led to our observed changes in behaviour, including: increased mass, increased drag forces, modifications to the centre of mass, or via the attachment procedure itself, which may induce stress and, in turn, lead to alterations in behaviour^38–42^. While the masses of our devices exceeded the 3% threshold^43^ upheld by some authors, the negative impacts associated with tagging have not been found to increase linearly with tag mass^11^, suggesting that a reduced mass threshold would not have been sufficient to eliminate the effects we observed here. Indeed, the attachment of tags < 3% of body mass has been found to cause negative impacts in a wide variety of species (e.g. great cormorant^18^; common starlings *Sturnus vulgaris*^44^; grey seal *Halichoerus grypus*^45^; Magellanic penguin *Spheniscus magellanicus*^46^; green turtle *Chelonia mydas*^47^; bottlenose dolphins^15^). In particular, the disruption of air or water flow associated with attaching an external tag means that the cost of movement can be increased substantially ^44,48,49^, especially for aquatic or volant animals. In addition to differences in the mass or impediment imposed by the different deployments discussed here, there are small differences in the duration and type of handling experienced by birds, so it is possible that these also contributed to the observed effects. However, any such effects are unlikely to be a main driver of our observed results. All birds, even those which were untagged, experienced handling, which was always short, and during deployments we did not observe visible signs of stress such as vocalisation or escape behaviour. The fact that we found no differences in foraging trip duration between GLS birds which had experienced occasional or frequent handling also supports this. Responses to handling have been shown to be less disturbing than device deployment in other seabirds^50^, and several studies in other Procellariiformes have shown that heart rate increases caused by handling return to base levels within a few hours^51,52^. It is therefore likely that in our study, the effects of handling dissipated quickly, and that the longer-term behavioural changes in flight and foraging behaviour we observed were driven by the persistent effects of carrying the device.

This may be the case in Manx shearwaters, which exhibited reduced flight time when carrying a GPS. It is unlikely that the birds in this study are physically prevented from ranging to long distance, profitable sites: while it is not possible to compare with untracked controls, GPS-tracked Manx shearwaters forage at considerable distances from the colony, covering the length of the Irish sea (approximately 280 km^40^), with trips averaging a total length of 1517 km (maximum 2117 km^1^). However, if increased flight costs compromise competitive ability, tracked birds may be competitively displaced from profitable patches, as may be the case for immature shearwaters^53^. Alternatively, the reduction in flight time observed may reflect a decrease in foraging efficiency associated with tagging. While tagged shearwaters in our study did not alter the amount of time they dedicated to foraging, it is possible that they increased foraging effort within this time. If shearwaters operate under an intrinsic energy ceiling^49^, and GPS-tracked birds invest more time in high-energy foraging, it may be the case that this increased energetic expenditure must be traded-off against reduced flight behaviour (energetically expensive) and increased rest (energetically inexpensive). Finally, while the change in rest time for GPS-tracked birds was not found to be significant, it is possible that the marginal increase in resting behaviour could reflect time 'wasted' pecking or preening at the tag^21^, with flight reducing as a consequence. If this is the case, individuals may be expected to habituate to tags over longer or multiple deployments, and so eventually return closer to baseline behaviour, though we did not have sufficient repeated measures of individuals to examine this in our dataset. Regardless of the specific mechanisms, we find that foraging gains per day are reduced. That the foraging trip duration overall is substantially increased in GPS-tracked individuals may reflect birds attempting to compensate for compromised foraging ability by extending the length of the trip itself. Consequently, the behaviour of GPS-tagged shearwaters is unlikely to be faithfully representative of unmanipulated individuals.

Despite this substantial disruption to behaviour, we did not observe changes in breeding success which could be related to device deployment type. This may in part reflect compensation by the partner. It is well established that in many species (e.g. Kentish plover *Charadrius alexandrinus*^54^; burying beetle^55^; starling^56^), including seabirds (e.g. Cape gannet *Morus capensis*^57^; great frigatebird *Fregata minor*^58^; Cory’s shearwater *Calonectris borealis*^59^), a reduction in care by one parent can be compensated by the partner. It is possible in this case that tagging disrupts the normally equally shared^60^ parental care burden. If the costs of tagging during chick rearing are absorbed by the un-tagged parent, then overall breeding outcome may not give an accurate assessment of tagging impacts in this species. This has been observed in thick-billed murres^38^ and black-legged kittiwakes (*Rissa tridactyla*)^61^, where a reduction in provisioning rate by tagged adults appears to be compensated by the partner, leading to normal fledging success. The stark differences in impact we observed between the two halves of the breeding season may therefore reflect the fact that during incubation we can measure precisely individual investment, while during chick-rearing we only measure the response of the pair as a whole, meaning compensation could occlude finer scale impacts on individual behaviour. It will therefore be important for future work to test whether nest visitation and feed delivery differs between GPS-tracked and untracked parents. Individual costs may additionally be absorbed into future reproductive attempts, as has been observed in northern wheatears (*Oenanthe oenanthe*^28^)*.* In Manx shearwaters, an experimental increase in parental effort in one year has been found to lead to reduced breeding success in the following year^62^, whilst natural release from breeding costs in one year (through breeding failure or by skipping breeding altogether) leads to improved breeding success in the subsequent year^63^. Consequently, long-term assessment of breeding success may be required to identify costs to reproduction. In long-lived species that produce few offspring, breeding success at a single point in time is unlikely to capture the full breadth of impact on an instrumented bird sufficiently. In our study, even major changes to individual behaviour, here identified as significant disruption to behaviour when foraging, were not reflected in breeding outcomes as we measured them. These results demonstrate that focusing on breeding success is inadequate to identify the full complement of impacts experienced by animals carrying tags.

It is now well understood that the instrumentation of wild animals can lead to undesirable effects on their fitness and behaviour. However, it is less clear how well fitness can provide a proxy for behavioural changes. Here, we identify major alterations to foraging behaviour which are not reflected in our measures of breeding success, highlighting the critical need for alternative measurements when considering the impacts of tagging. It is likely that the disjunction between effects on fitness and behaviour observed here are commonplace in instrumented species. Consequently, careful consideration of which impacts are of interest from the perspective of the study design are necessary to determine whether data collected are representative of the wider population. By considering tagging impacts beyond coarse measures such as breeding success in a single season, authors can better identify from where their impacts arise and hence attempt to mitigate them. Even simple measures, such as comparisons of individual condition (easily measured as mass) or assessments of behavioural measures (such as trip length), in comparison to controls, will give a more complete picture of the often complex impacts of tag deployment on individuals, ultimately giving us a better toolkit with which we can answer fundamental questions about animal behaviour.

## Methods

### Study site and species

The study was conducted from 2009 to 2017 on UK Manx shearwater breeding colonies and long-term studies sites at Skomer Island, Wales (51°44′ N, 5°17′ W) and Lighthouse Island in the Copelands group, Northern Ireland (54°44′ N, 5°31′ W). Manx shearwaters breed on dense island colonies between April and September, during which time they come onto land only at night. In April, females lay a single egg in an underground burrow, which is incubated for about 51 days, with stints of 5–7 days taken by each parent. Following hatching, both parents feed the chick most nights for approximately 60 days. Occupancy and breeding success were monitored at the Skomer colony each year, with birds on both islands being easily accessed at the nest through the burrow entrance or purpose-built inspection hatches. To allow individual identification, all birds in this study were ringed with a permanent stainless steel ring, provided by the British Trust for Ornithology. Females were identified at the point of laying through cloacal inspection^64^, and males by inference.

### Sampling methods

GPS tracking campaigns lasted for approximately two weeks during the incubation (May–June) and chick rearing periods (July–August). Deployments on the two islands were carried out simultaneously in each given year. We analysed the foraging trip metrics during incubation and provisioning behaviour of birds subject to four different logger deployments: no device (ring only, 0.78 g), a geolocator only (2.5 g), a single global positioning system (GPS) logger (total attachment mass 17 g), or a GPS logger and geolocator (GLS, c. 19.5 g). The sample sizes in each of these groups can be found in Table 4.

**Table 4:** Sample size (summed across years) for each deployment type.

No device	GLS	GPS		Combined	
		Inc	CR	Inc	CR
85	106	59	56	181	54

*Inc* = incubation period, *CR* = chick-rearing period.

For individuals carrying no logger, foraging trips were identified by directly identifying the incubating parent once a day from laying to hatching, by removing the incubating adult from the nest and reading its ring number, which took no longer than 1 min, adding to a total of c. 20 min over the whole incubation period. Whichever bird was not present at the nest was deemed to be on a foraging trip.

GLS (Migrate Technology Intigeo-C250 or Intigeo-C65; BioTrack Mk4083; British Antartic Survey MK-14 or MK-19), were deployed either simultaneously with GPS (combined group) or at the beginning of the breeding season (GLS group), in April, and were retrieved at the beginning of the breeding season the following year. GLS were attached by two small cable ties to a plastic leg ring to ensure immersion when sitting on the sea (see^65^). Deployment and retrieval of GLS was conducted in the field and handling time did not normally exceed 5 min. 27 individuals carrying GLS were handled daily, as per the no device birds, as part of a separate experiment. We analysed whether these two subgroups differed in their mean foraging trip duration; as they did not, we pooled the subgroups for the remainder of the analysis (see supplementary materials). GPS loggers (I-gotU GT-120) were waterproofed using lightweight heat-sealed plastic sleeves and attached to the dorsal feathers of birds using 5 strips of marine TESA tape (see^40^ for details). Birds were captured on the nest during changeover with the partner (egg incubation) or during a provisioning visit to the nest (chick rearing), after they had been allowed approximately 30 min to feed their chick. GPS deployment was carried out in a darkened lab and processing time was approximately 10 min. On both islands the lab is close to the colony and so transportation did not exceed 10 min, giving a total maximum handling time, from capture to release, of 20 min. Following deployment of the GPS, the bird was returned to the nest and allowed to depart on its foraging trip naturally. Incubating birds were recaptured as soon as they returned from foraging; efforts to recapture chick-provisioning birds began 3 days after departure until their subsequent return to the nest. The foraging trip duration of incubating birds could thus be identified as the period it was not found in the nest.

### Mass changes and breeding success

To measure foraging gains from each trip, mass measurements were taken for all GPS birds at deployment and retrieval, and were taken daily during direct observations of non-instrumented and 23 GLS-tracked nests made during incubation in 2015 and 2016. Birds were inserted head first into a draw-string muslin bag attached to a 600 g Pesola spring balance (precise to 5 g). The resulting mass change (mass at return – mass at departure) was divided by the duration of the foraging trip (in days) to give a measure of daily mass gain.

To determine the meal sizes provided by parents, chick masses were collected on Skomer in 96 nests where at least one parent carried a GPS, 48 where at least one carried a GLS, and 89 where neither parent carried a device, between 2013 and 2017. Chicks were placed into a plastic box and weighed on a digital balance precise to 1 g. Chick masses on Skomer were collected daily from the day following hatching until the chick was not found in the nest for 3 consecutive days, at which point it was presumed to have fledged. For each nest, the duration of the provisioning period was identified as the time between hatching and the last known feed (taken to be an increase in the chick's body mass). For those chicks surviving to fledgling stage, the maximum mass attained, total number of nights fed, and average daily mass change was calculated.

At the end of the season, data on breeding outcome were collected for each nest in the Skomer study colony. Breeding outcomes were not available for Copeland birds.

### Extracting foraging trip metrics

As foraging trip durations were not available for unmanipulated or GLS-carrying birds during chick-rearing, differences in foraging trip metrics were only investigated during the incubation period. For each year and tracking campaign, only foraging trips from GLS-only and no device birds that occurred simultaneously with the GPS-tracking period were used in the analysis. GLS devices tested for saltwater immersion every 3 or 6 s and recorded the proportion of samples immersed in water in each 10- or 5-min epoch respectively. The frequency distributions of total daily immersion (sum of each 10- or 5- minute immersion score) recorded by the GLS were used to separate incubation stints from foraging trips, using the expectation that days of incubation in the burrow would be characterized by a distribution of very low total immersion values. The package mixtools^66^ was used to fit a Gaussian mixture model to the frequency data, which was used to assign days with lower total immersion as incubation days. These assignments were subsequently validated using individuals for which we knew through direct observation whether the bird was incubating or not. This yielded a 92% accuracy for assignment of foraging dates.

At-sea behaviour was classified using immersion data, using the same threshold methods outlined by Fayet et al., 2016^67^, where classifications were outlined as: < 2% maximum immersion for directed flight, > 98% maximum immersion for resting on the water, and intermediate values for foraging. Intermediate values are caused by the bird taking off and landing on the water, and have been shown to be indicative of foraging behaviours in this species by validation with dive loggers^1^.

### Statistical analysis

All statistical analyses were completed using R version 3.2.2^68^. The R package lme4^69^ was used to construct linear mixed effects models (LMMs), and p- values were obtained by comparing models to null models without the effect of interest, using a likelihood ratio test. Least squares means for each level of the deployment type were calculated using the R package emmeans^70^, and Tukey’s range test used to calculate significant differences between them. To ensure that repeated measurements from the same individuals or inter-year differences did not impact our parameter estimates, all models included a random intercept effect of individual nested within year. For our model of breeding success, the random intercept was burrow identity nested within year.

To investigate differences in breeding success according to deployment type, a generalized linear mixed effects model with a binomial distribution was fitted to breeding success in the current (*t*) and subsequent (*t* + *1*) year. Breeding success in *t* + *1* additionally included the fixed effect of breeding success in *t*.

To ensure that differences in trip length could not be explained by differences in the starting masses of birds, an LMM was fitted to determine the effect of deployment type (no device, GLS, GPS or combined) on (1) starting mass (g). Further LMMS were fitted to examine the effect of deployment type on (2) foraging trip duration, (3) daily foraging gain, and (4) total foraging gains. Models 2–4 included the fixed effect of starting mass (g) and sex; model 2 additionally included the interaction of deployment type and body condition was as a fixed predictor. To allow for an increased sample size and greater variation in foraging trip duration across the treatments, foraging trips from both Copeland and Skomer birds were included in the analysis. As mean foraging trip duration differs between the two islands, island was included as a fixed effect in all 3 models. For data collected during the chick rearing period, LMMS were also fitted to (5) provisioning trip duration, (6) maximum chick mass obtained, (7) daily feed size, and (8) number of nights fed. Models 5–8 included the random effect of nest within year to control for repeated measurements from nests.

To investigate differences in at-sea behaviour during foraging trips for birds in the GLS and combined deployment groups, LMMs were fitted to determine the effect of deployment type on the proportion of time spent in each of foraging, resting, and flight states per day of the trip. Trip duration and island were additionally included as fixed effects.

### Ethical note

All methods and procedures adhere to ASAB/ABS Guidelines for the Use of Animals in Research, and were approved by the British Trust for Ornithology (BTO) Unconventional Methods Technical Panel (permit number C\5311) and by the Wildlife Trust for South and West Wales under the name of Prof. Tim Guilford. Ethical approval was received from the Local Ethical Review Process of the University of Oxford. This project is covered by Northern Ireland Environment Agency (NIEA) permits, and holds Islands Conservation Advisory Committee (ICAC) approval. To reduce the potential for stress, handling time was kept to a minimum.

## Supplementary information

Supplementary Information.
