# Supplementary material for: Short-term behavioural impact contrasts with long-term fitness consequences of biologging in a long-lived seabird
Source: Sci Rep. 2020 Sep 14;10:15056. doi: 10.1038/s41598-020-72199-w (PMC7490266; doi:10.1038/s41598-020-72199-w)
Supplement: Supplementary file 1 — Supplementary Information. [file 41598_2020_72199_MOESM1_ESM.pdf]

Supplementary Materials: Short-term behavioural impact contrasts with long-term fitness consequences of biologging in a long-lived seabird

Natasha Gillies[a]*, Annette L. Fayet[a], Oliver Padget[a], Martyna Syposz[a], Joe Wynn[a], Sarah Bond[b], James Evry[a], Holly Kirk[c], Akiko Shoji[d], Ben Dean[a], Robin Freeman[e], Tim Guilford[a]*

[a]Department of Zoology, University of Oxford, United Kingdom

[b]School of Ocean Sciences, Bangor University, United Kingdom

[c]Interdisciplinary Conservation Science Group, RMIT University, Australia

[d]Graduate School of Life and Environmental Sciences, University of Tskuba, Japan

[e]Institute of Zoology, Zoological Society of London, United Kingdom

Starting masses by deployment group

No device: 387.8 ± 5.6g, GLS: 399.7 ± 6.8g, GPS: 400.2 ± 4.9g, combined: 401.7 ± 3.4g

$\chi^2 = 6.9$, df = 3, p = 0.074

Foraging trip length according to island

Copeland: 4.06 ± 0.7 days, Skomer: 8.5 ± 0.5 days

$\chi^2 = 109.7$, df = 1, $p < 0.0001$

Foraging trip duration by deployment group

No device: 4.8 ± 0.5 days, GLS: 5.2 ± 0.4 days, GPS: 9.4 ± 0.5 days, combined: 8.9 ± 0.4 days

$\chi^2 = 182.4$, df = 3, $p < 0.0001$

There was no significant effect of the interaction of deployment type and starting mass ($\chi^2 = 0.8$, df = 3, p = 0.8).

At-sea activity by deployment group

Proportion of time in flight

GLS: 0.13 ± 0.008, combined: 0.11 ± 0.01

$\chi^2 = 6.2$**,** df = 1 p = 0.01

Proportion of time in resting state

GLS: 0.37 ± 0.01, combined: 0.40 ± 0.01

$\chi^2 = 6.0$, df = 1, p = 0.01

Proportion of time spent foraging

GLS: 0.50 ± 0.01, combined: 0.49 ± 0.01

$\chi^2 = 2.4$, df = 1, p = 0.1

Mean daily foraging mass gain by deployment group

No device: 8.8 ± 0.8g, GLS: 9.4 ± 1g, GPS: 2.7 ± 0.8g, combined: 4.7 ± 0.5g

$\chi^2 = 45.9$, df = 3, p < 0.0001

Total foraging trip mass gain by deployment group

No device: 58.7 ± 5.0g, GLS: 66.0 ± 5.9g, GPS: 30.7 ± 4.9g, combined: 39.9 ± 3.3g

$\chi^2 = 26.88$, df = 3, p < 0.0001

Supplementary Table S1. Pairwise comparison of foraging trip duration during incubation for each deployment type. Significant values (p < 0.05) are in bold.

Device A	Device B	Mean diff (A - B)	t value	p
No device	GLS	-0.4 ± 0.4	-0.9	0.779
	**GPS**	**-4.6 ± 0.5**	**-9.3**	**< 0.0001**
	**Combined**	**-4.1 ± 0.4**	**-9.8**	**< 0.0001**
**GLS**	**GPS**	**-4.2 ± 0.5**	**-9.0**	**< 0.0001**
	**Combined**	**-3.7 ± 0.3**	**-11.4**	**< 0.0001**
GPS	Combined	0.4 ± 0.5	1.0	0.773

Discerning the Impacts of Handling

To attempt to investigate whether differences in handling time could have precipitated the observed behavioural changes between deployment groups, we compared the foraging trip durations of GLS tracked birds which were handled daily (high intensity handling) or only once prior to data collection, for deployment of the GLS device (low intensity handling). We constructed a LMM which examined foraging trip duration as a function of handling intensity, island, and sex, and included a random intercept of ring number nested within year. As there were no differences in trip duration between these two subgroups, they were combined for all other analyses.

For GLS-tracked birds, differences in handling intensity (daily vs once for deployment) were not associated with differences in foraging trip duration (low intensity handling: 5.9 ± 1.2 days, high intensity handling: 6.4 ± 1.3 days; df = 1, $\chi^2 = 1.1$, p = 0.3).
